# Supplementary material for: RNA degradation triggered by decapping is largely independent of initial deadenylation
Source: EMBO J. 2024 Sep 25;43(24):6496–524. doi: 10.1038/s44318-024-00250-x (PMC11649920; doi:10.1038/s44318-024-00250-x)
Supplement: Supplementary file 1 — Appendix [file 44318_2024_250_MOESM1_ESM.pdf]

Appendix

RNA degradation triggered by decapping is largely independent of initial deadenylation

Appendix Table S1 - page 1

Appendix Figure S1 - Robustness of the negative correlations between poly(A) tail length and half-life of RNA. - page 2

Appendix Figure S2 - Model-based scenarios for predicted effects of slowing down deadenylation and decapping. - page 4

Appendix Figure S3 - Correlation of SLAM-Seq results with previous mRNA half-life estimates. - page 5

Appendix Table S1. HIS3 coding sequence for reporters (non-OPT/OPT)

non-OPT-HIS3 coding region HIS3 sequence
ATGGACTACAAGGACGACGACGACAAGACAGAGCAGAAAGCACTAGTAAAGCGGATAACAAATGAGACAAAGATTCAGATAGCGATAA<br>GTTTAAAAGGAGGTCCCCTAGCGATAGAGCATTCGATATTTCCCGAGAAAGAGGCAGAAGCAGTAGCAGAGCAGGCAACACAGTCGCA<br>AGTGATAAATGTGCATACAGGAATAGGGTTTCTGGATCATATGATACATGCGCTGGCAAAACATTCGGGCTGGTCGCTAATAGTGGAG<br>TGCATAGGAGATTTACATATAGATGATCATCACACAACAGAGGATTGCGGGATAGCTCTCGGTCAAGCATTTAAAGAGGCGCTAGGGG<br>CAGTGCGTGGAGTAAAAAGGTTTGGATCAGGATTTGCGCCTTTGGATGAGGCACTTTCCAGAGCGGTGGTAGATCTTTCGAACAGGCC<br>GTATGCAGTTGTGGAGCTTGGCCTCCAGAGGGAGAAAGTAGGAGATCTCAGTTGCGAGATGATACCGCATTTTCTTGAGAGCTTTGCA<br>GAGGCGAGCAGGATAACCCTCCATGTGGATTGCCTGCGAGGCAAAAATGATCATCATCGGAGTGAGAGTGCGTTTAAAGCTCTTGCGG<br>TAGCAATACGCGAGGCCACATCGCCCAATGGCACCAATGATGTTCCCTCGACAAAAGGGGTACTTATGTAG
OPT-HIS3 coding region HIS3 sequence
ATGGACTACAAGGACGACGACGACAAGACTGAACAAAAGGCCTTGGTTAAGCGTATTACTAACGAAACCAAGATTCAAATTGCCATCT<br>CTTTGAAGGGTGGTCCATTGGCCATTGAACACTCCATCTTCCCAGAAAAGGAAGCTGAAGCTGTTGCTGAACAAGCCACTCAATCCCA<br>AGTCATTAACGTCCACACTGGTATTGGTTTCTTGGACCACATGATTCACGCTTTGGCCAAGCACTCCGGTTGGTCCTTGATCGTTGAA<br>TGTATTGGTGACTTGCACATTGACGACCACCACACCACTGAAGACTGTGGTATTGCTTTGGGTCAAGCTTTCAAGGAAGCCTTGGGTG<br>CCGTCCGTGGAGTAAAAAGGTTTGGATCAGGTTTCGCCCCATTGGACGAAGCTTTGTCCAGAGCCGTCGTTGACTTGTCCAACAGACC<br>ATACGCTGTTGTCGAATTGGGTTTGCAAAGAGAAAAGGTTGGTGACTTGTCTTGTGAAATGATCCCACACTTCTTGGAATCCTTCGCT<br>GAAGCTTCCAGAATTACCTTGCACGTTGACTGTTTGCGTGGTAAGAACGACCACCACCGTTCTGAATCTGCCTTCAAGGCTTTGGCCG<br>TTGCCATTAGAGAAGCCACCTCCCCAAACGGTACCAACGACGTTCCATCCACCAAGGGTGTTTTGATGTAG

Appendix Supplementary Figures

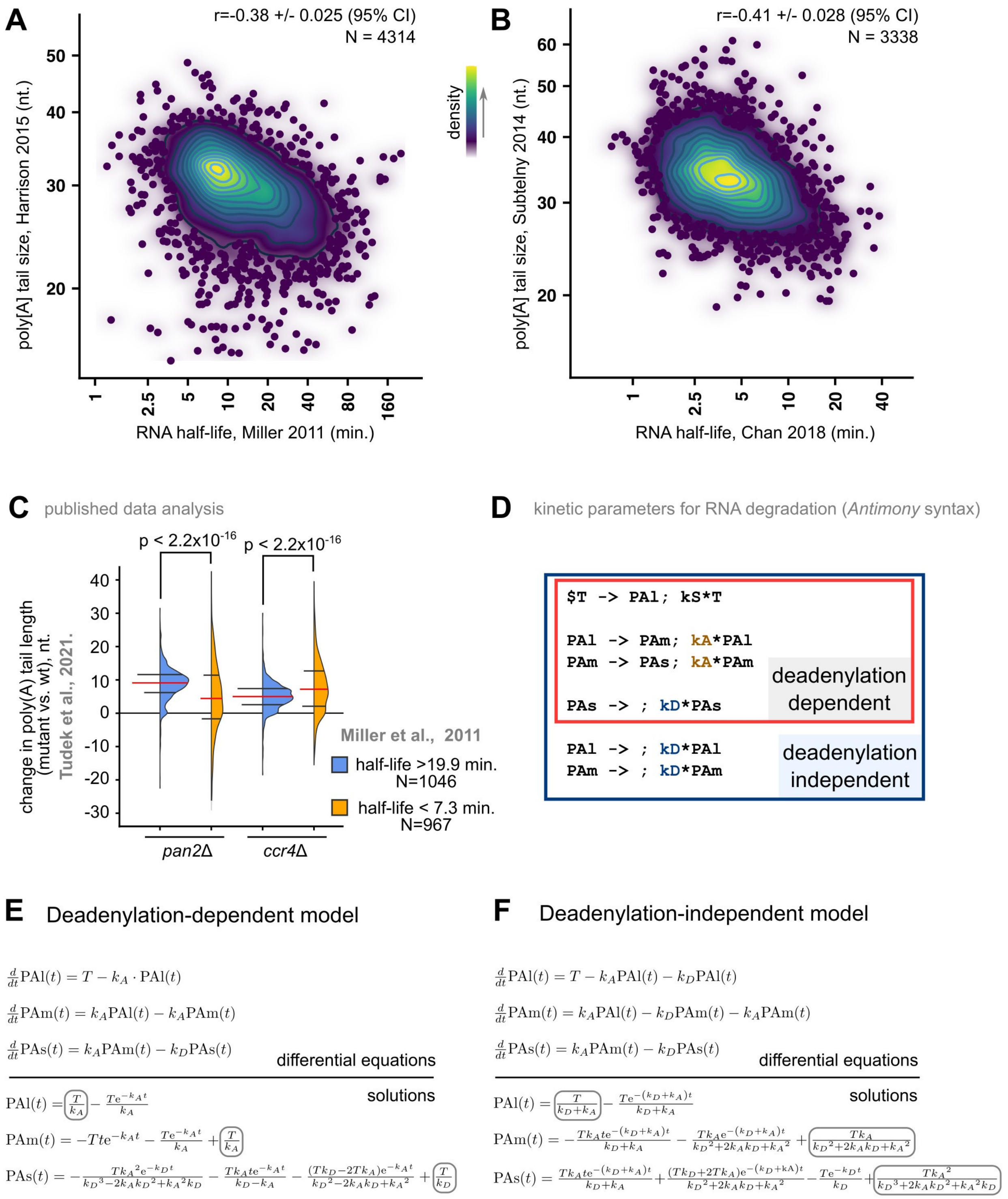

Appendix Fig. S1. Robustness of the negative correlations between poly(A) tail length and half-life of RNA. (A), (B) Negative correlations between published results, similar to the ones shown in Fig. 2A and 2B, including results for poly(A) tail size (Harrison *et al*, 2015; Subtelny *et al*, 2014) and

estimates of RNA half-life (Chan *et al*, 2018; Miller *et al*, 2011). **(C)** RNAs for which half life is short, in the first quartile, half life inferior to 7.3 min, or long, in the third quartile, over 19.9 min (Miller *et al*, 2011), were selected to evaluate how their poly(A) size changed in deadenylation mutants, as measured by Nanopore sequencing (Tudek *et al*, 2021). The quartiles of the distributions are indicated, median in red, as well as the p-value of a Wilcoxon rank sum test with continuity correction for the difference between the two populations. **(D)** Parameters used to simulate RNA degradation measurements that include poly(A) tail information and kinetic equations using the *Antimony* syntax (Smith *et al*, 2009). "T" represents all the processes leading to mRNA formation. PA_l, PA_m and PA_s represent concentrations of different poly(A) tail RNA species. **(E)** The set of differential equations used to estimate time changes for the three modeled poly(A) species in a deadenylation-dependent model (upper region) and the solutions (lower region). The terms that correspond to steady-state are circled in gray. **(F)** Similar to (E), for the deadenylation-independent model.

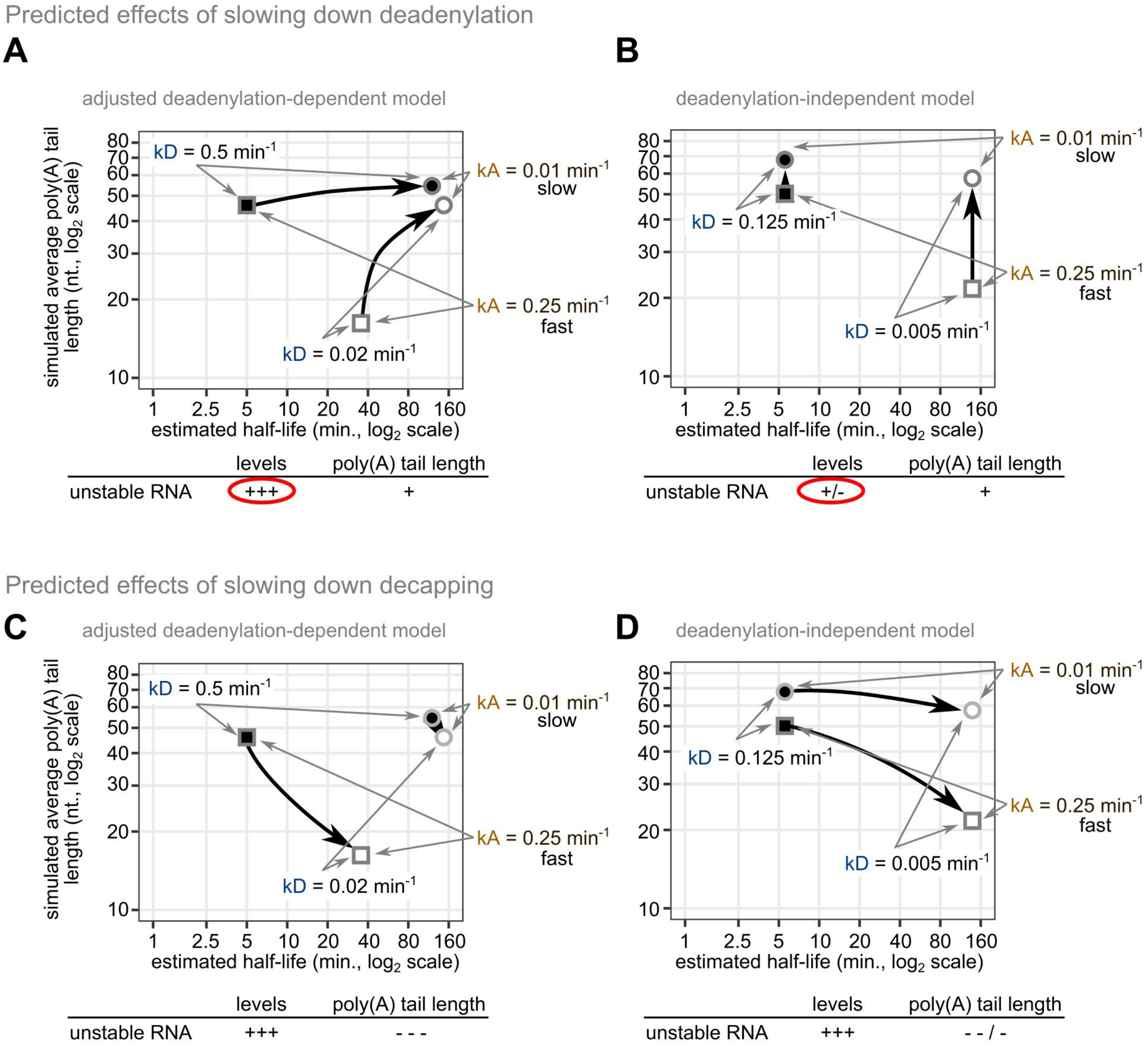

Appendix Fig. S2. Model-based scenarios for predicted effects of slowing down deadenylation (A, B) and decapping (C, D). For each situation, the expected shift in poly(A) tail length and stability for a stable and an unstable RNA are indicated by black arrows. The experimental conditions can only estimate changes in poly(A) tails and levels for relatively unstable mRNAs (situation depicted under each graph).

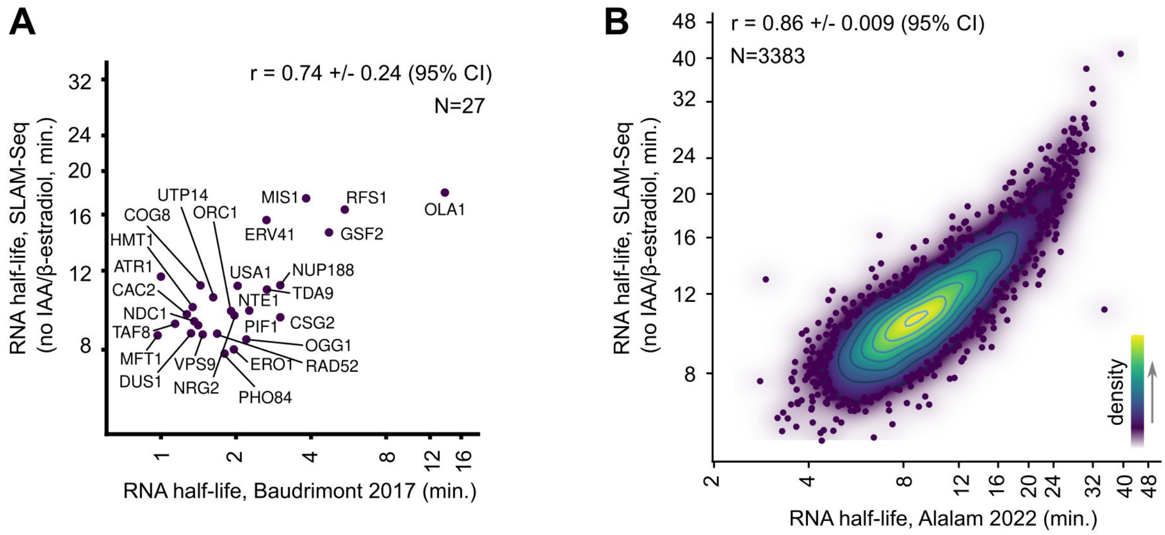

Appendix Fig. S3. Correlation of SLAM-Seq results with previous mRNA half-life estimates. (A) Comparison with individual results from Baudrimont et al. 2017. **(B)** Comparison with SLAM-Seq results of Alalam et al., 2022. In both panels, Pearson's product moment correlation coefficients and their 95% confidence intervals are indicated.
