# Supplementary figures and images for: RNA degradation triggered by decapping is largely independent of initial deadenylation
Source: EMBO J. 2024 Sep 25;43(24):6496–524. doi: 10.1038/s44318-024-00250-x (PMC11649920; doi:10.1038/s44318-024-00250-x)

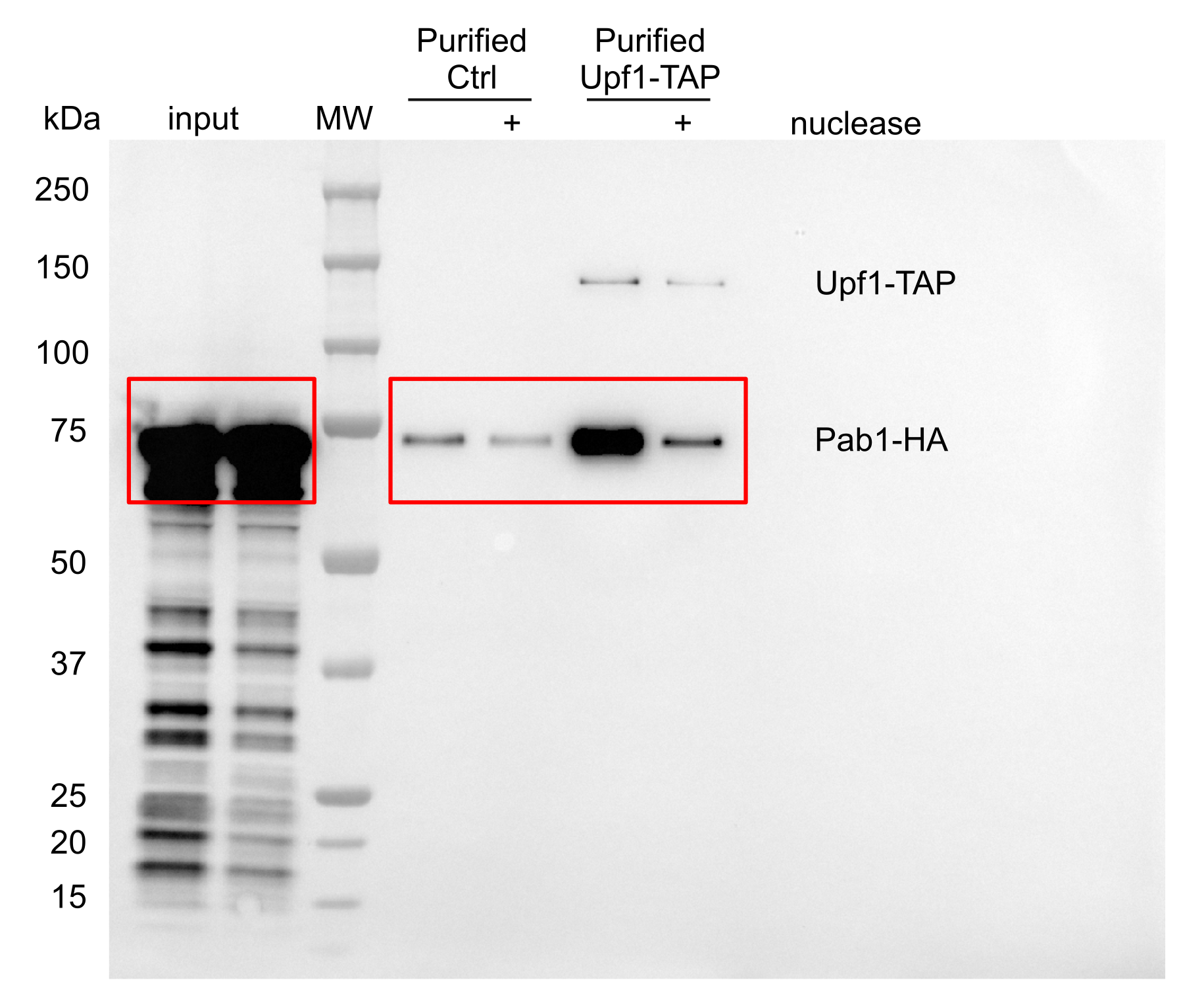

Supplement: Supplementary file 8 — Source data Fig. 1 [file 44318_2024_250_MOESM8_ESM.zip › Figure1/1B/immunoblot_Pab1_HA.tif]

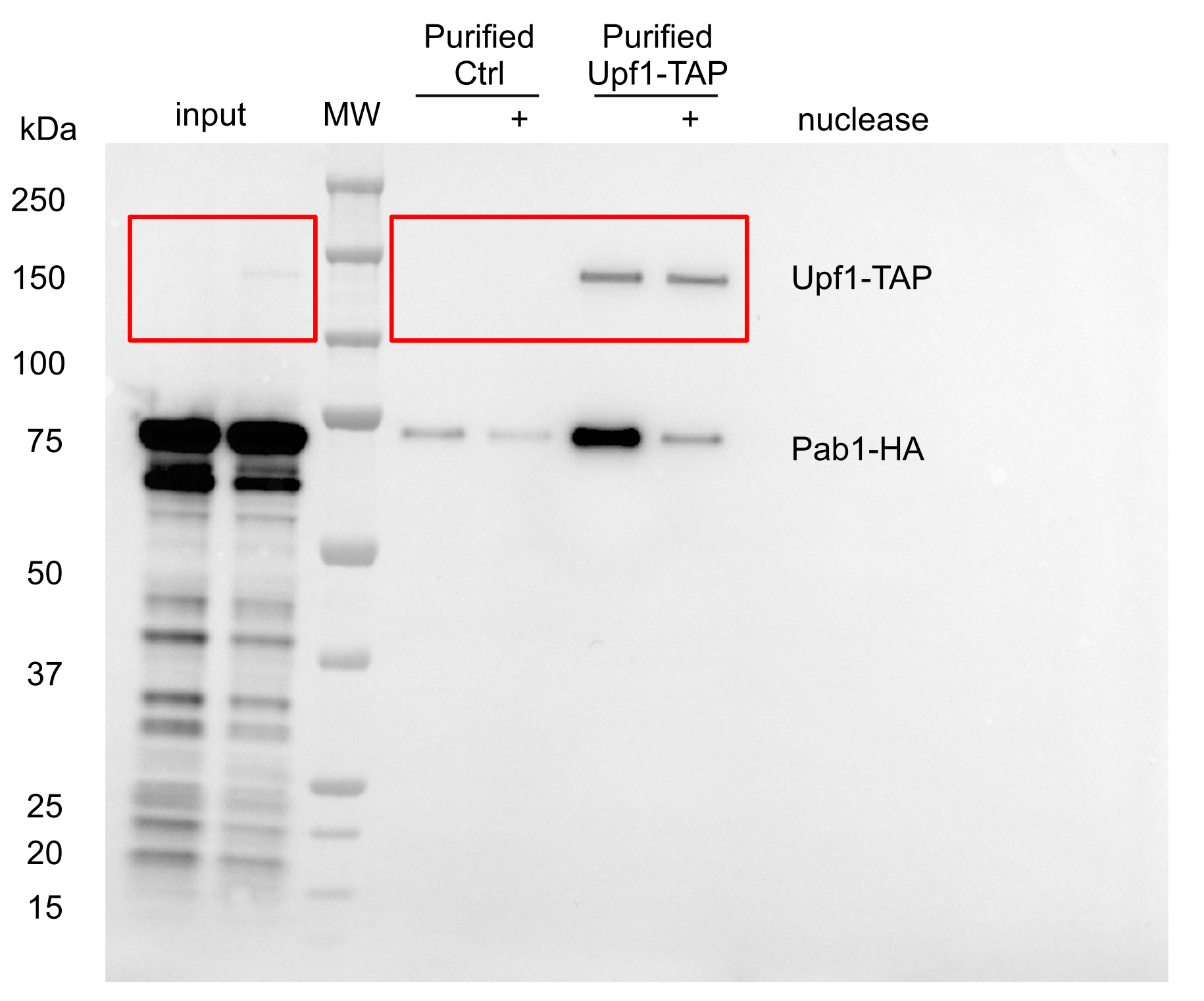

Supplement: Supplementary file 8 — Source data Fig. 1 [file 44318_2024_250_MOESM8_ESM.zip › Figure1/1B/immunoblot_Upf1_TAP.tif]

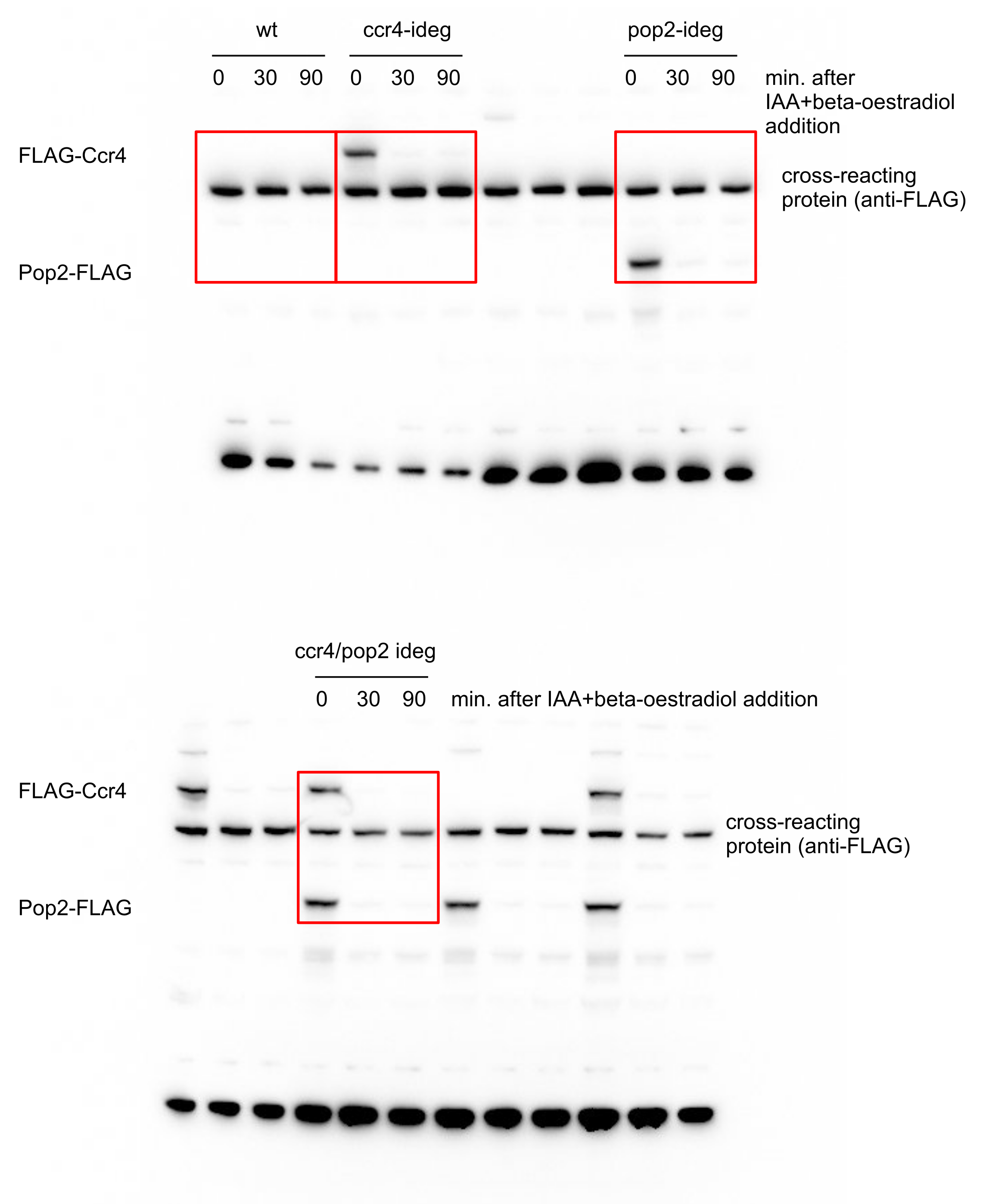

Supplement: Supplementary file 9 — Source data Fig. 3 [file 44318_2024_250_MOESM9_ESM.zip › Figure3/3A/immunoblot_ccr4_pop2_FLAG.tif]

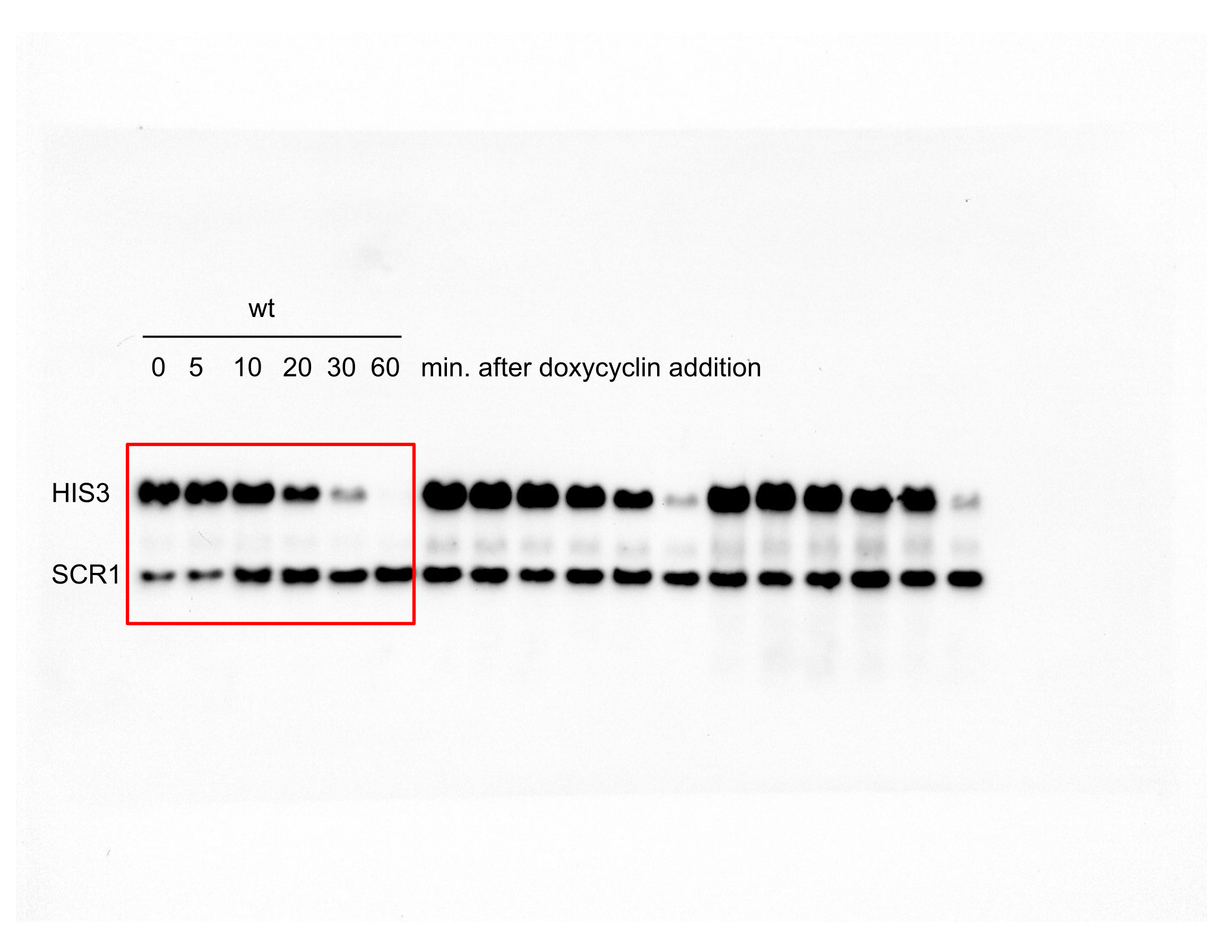

Supplement: Supplementary file 10 — Source data Fig. 5 [file 44318_2024_250_MOESM10_ESM.zip › Figure5/5B/OPT_HIS3_wt_northern_blot.tif]

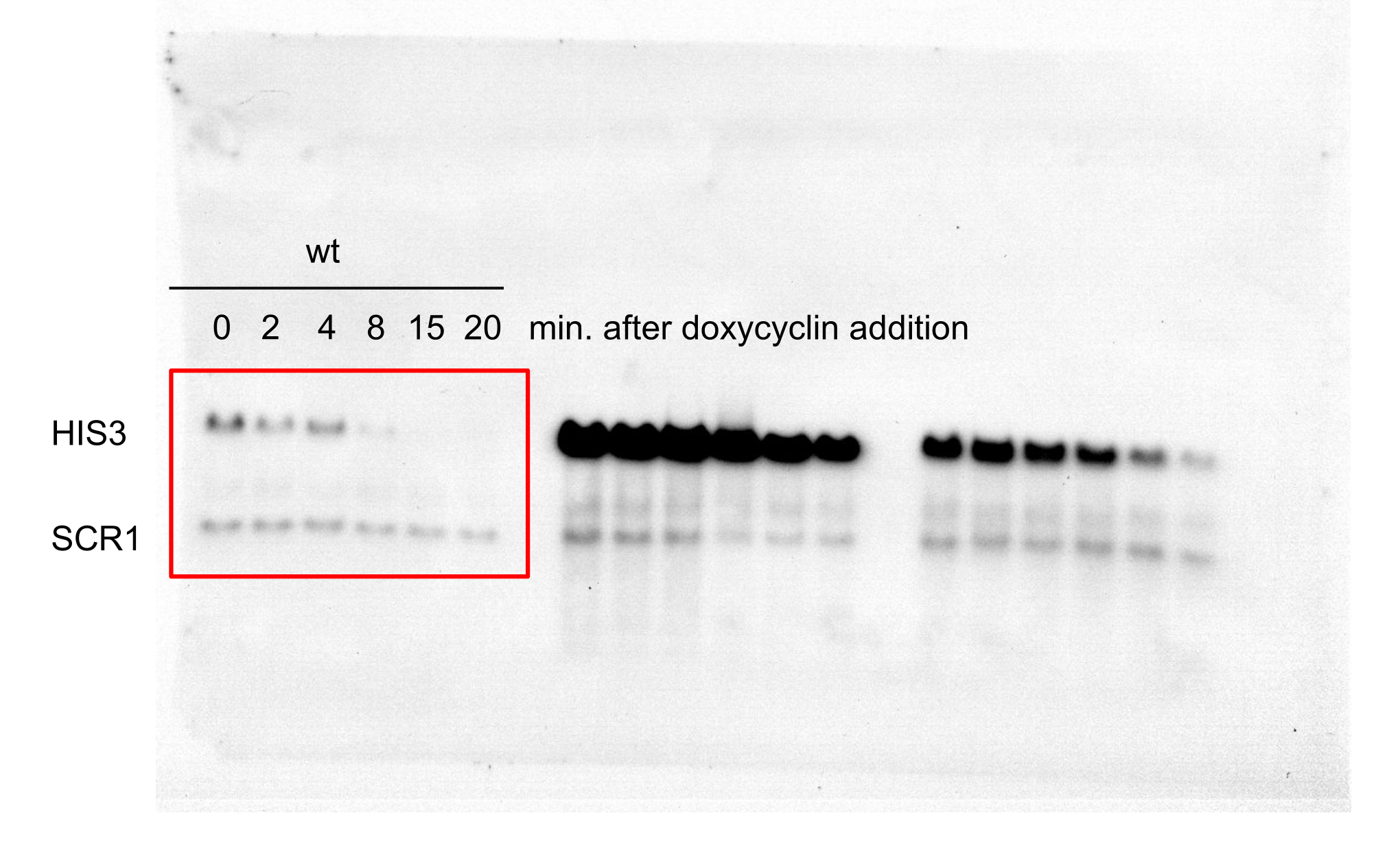

Supplement: Supplementary file 10 — Source data Fig. 5 [file 44318_2024_250_MOESM10_ESM.zip › Figure5/5B/nonOPT_HIS3_wt_northern_blot.tif]

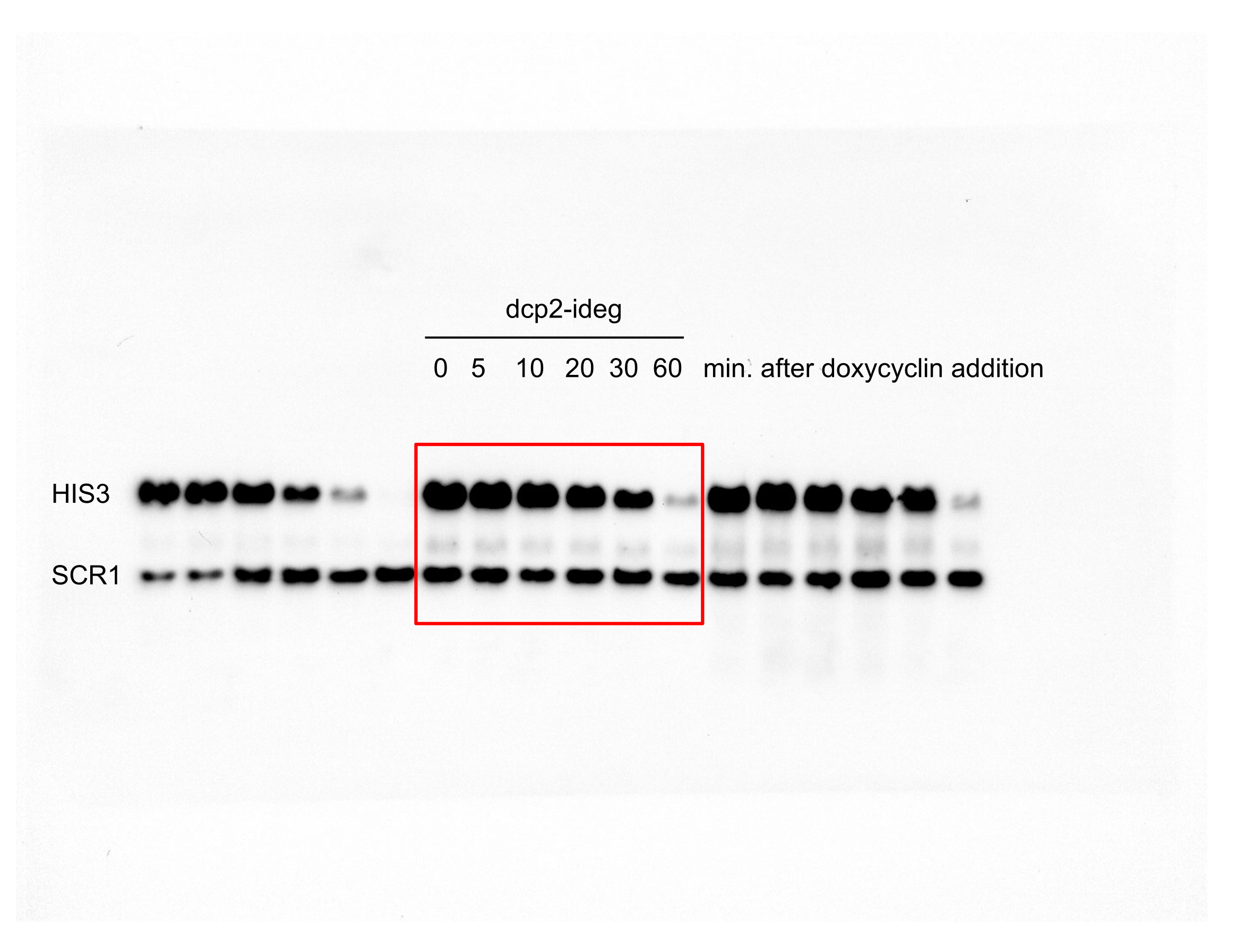

Supplement: Supplementary file 10 — Source data Fig. 5 [file 44318_2024_250_MOESM10_ESM.zip › Figure5/5C/OPT_HIS3_dcp2ideg_northern_blot.tif]

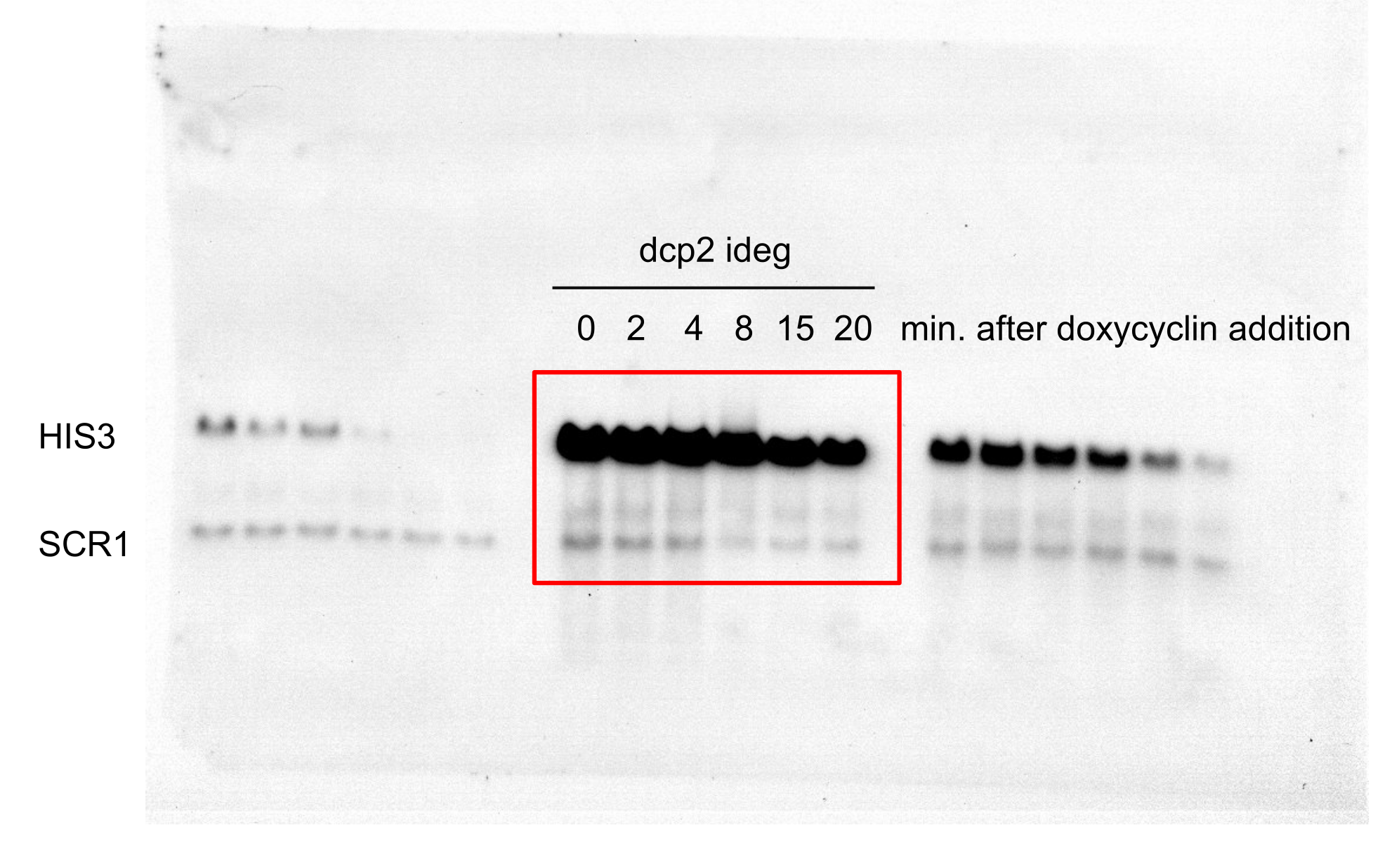

Supplement: Supplementary file 10 — Source data Fig. 5 [file 44318_2024_250_MOESM10_ESM.zip › Figure5/5C/nonOPT_HIS3_dcp2ideg_northern_blot.tif]

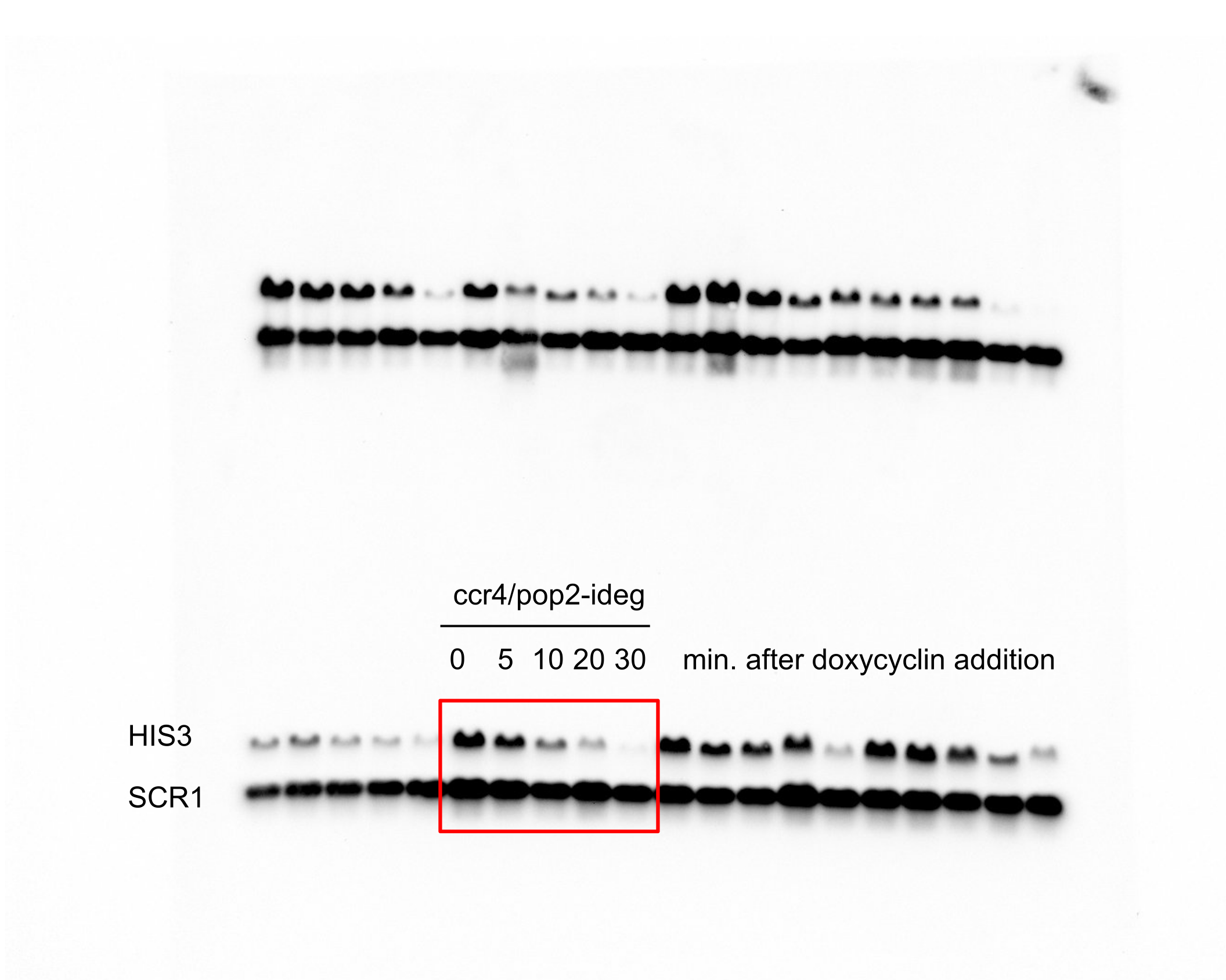

Supplement: Supplementary file 10 — Source data Fig. 5 [file 44318_2024_250_MOESM10_ESM.zip › Figure5/5D/OPT_HIS3_ccr4_pop2_northern_blot.tif]

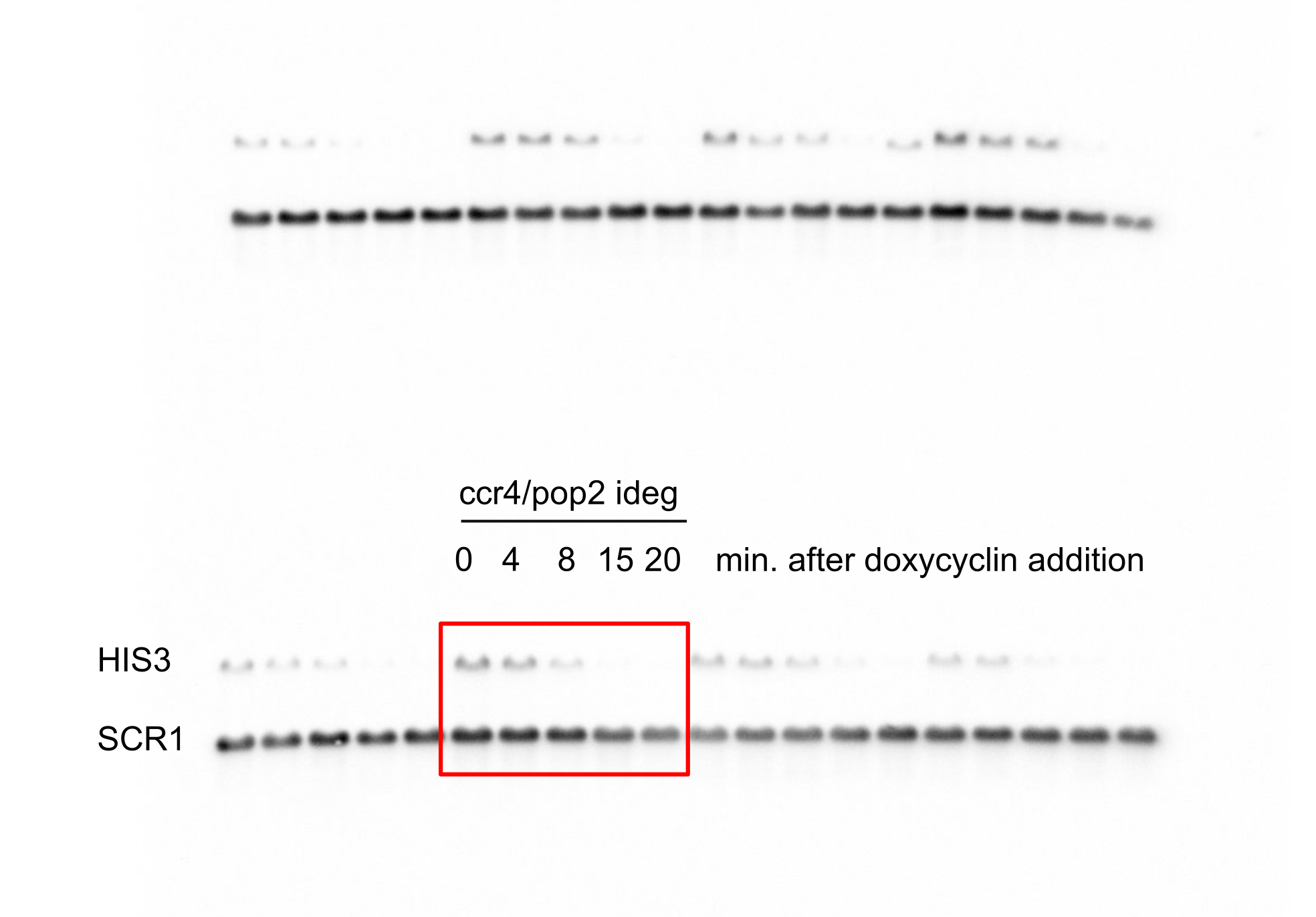

Supplement: Supplementary file 10 — Source data Fig. 5 [file 44318_2024_250_MOESM10_ESM.zip › Figure5/5D/nonOPT_HIS3_ccr4_pop2_northern_blot.tif]

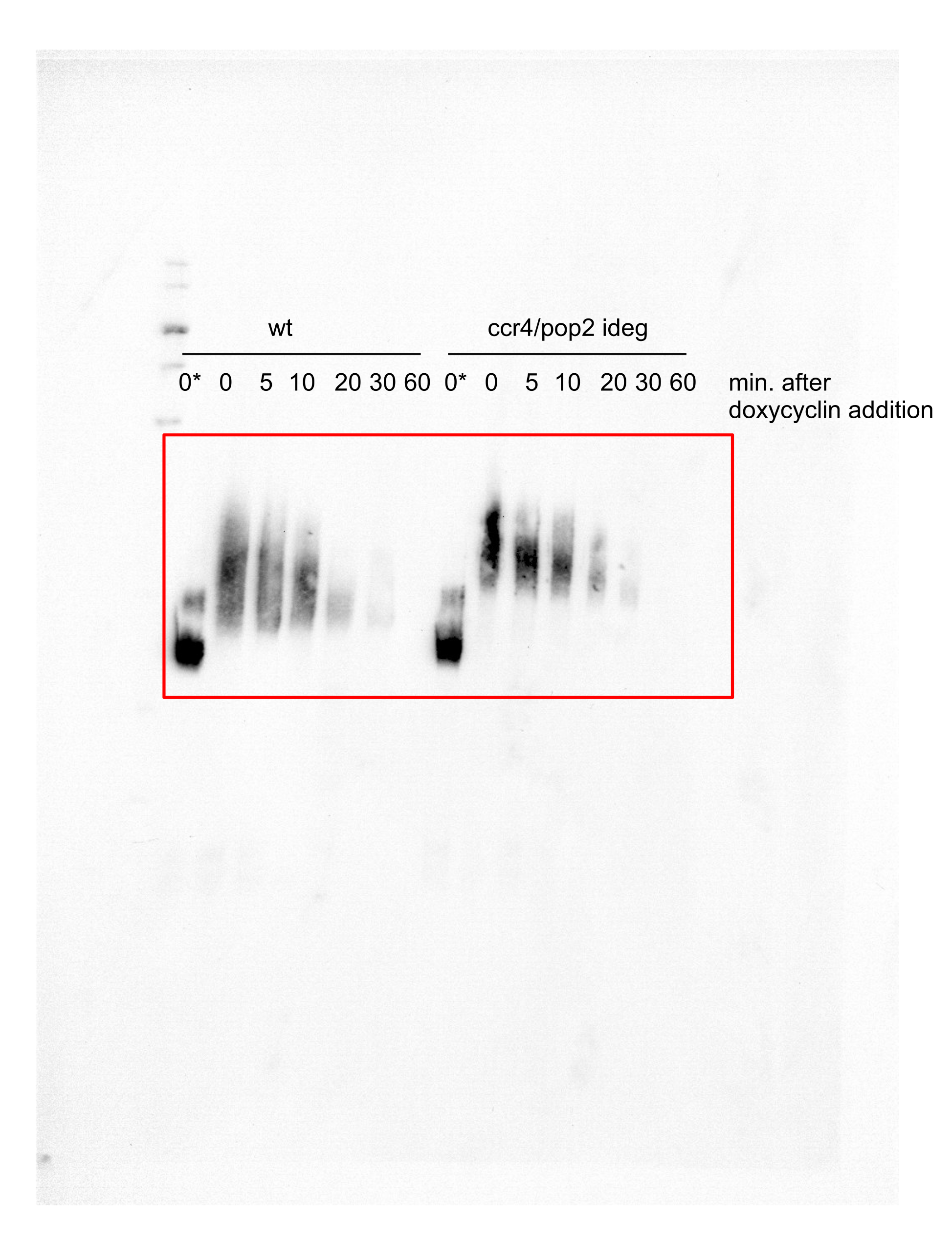

Supplement: Supplementary file 10 — Source data Fig. 5 [file 44318_2024_250_MOESM10_ESM.zip › Figure5/5E/RnaseH_Northern_blot_HIS3.tif]

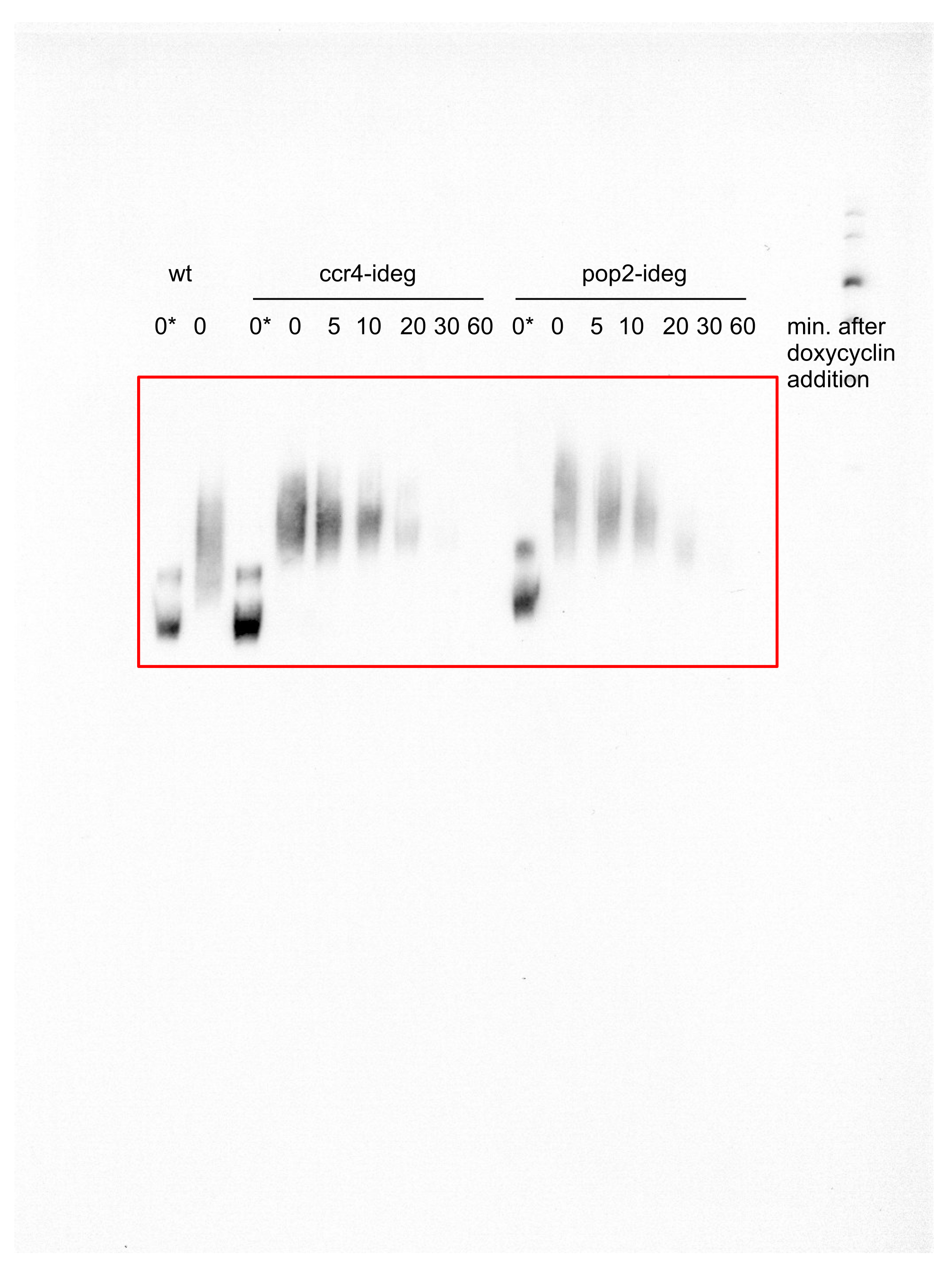

Supplement: Supplementary file 10 — Source data Fig. 5 [file 44318_2024_250_MOESM10_ESM.zip › Figure5/5F/RnaseH_Northern_blot_HIS3.tif]
