# Supplementary material for: RNA degradation triggered by decapping is largely independent of initial deadenylation
Source: EMBO J. 2024 Sep 25;43(24):6496–524. doi: 10.1038/s44318-024-00250-x (PMC11649920; doi:10.1038/s44318-024-00250-x)
Supplement: Supplementary file 11 — Expanded View Figures [file 44318_2024_250_MOESM11_ESM.pdf]

Expanded View Figures

Figure EV1. Enrichment of specific factors in purified complexes containing Upf1.

Volcano plots represent the enrichment of proteins, relative to the protein abundance in a total extract as measured by label-free mass spectrometry. Results are presented for purifications using TAP tagged versions of Lsm1 (**A**), Pat1 (**B**), Lsm7 (**C**), and Dhh1 (**D**). Ribosomal proteins are highlighted in blue. (**E**) Examples of enrichment values across the various purifications for Lsm2, a component of Lsm1-7 and Lsm2-8 complexes, and Upf1. Error bars represent standard deviation. *N* is 3 for Dhh1, Lsm1, Lsm7, and Pab1 purifications and 4 for Pat1.

 © The Author(s)

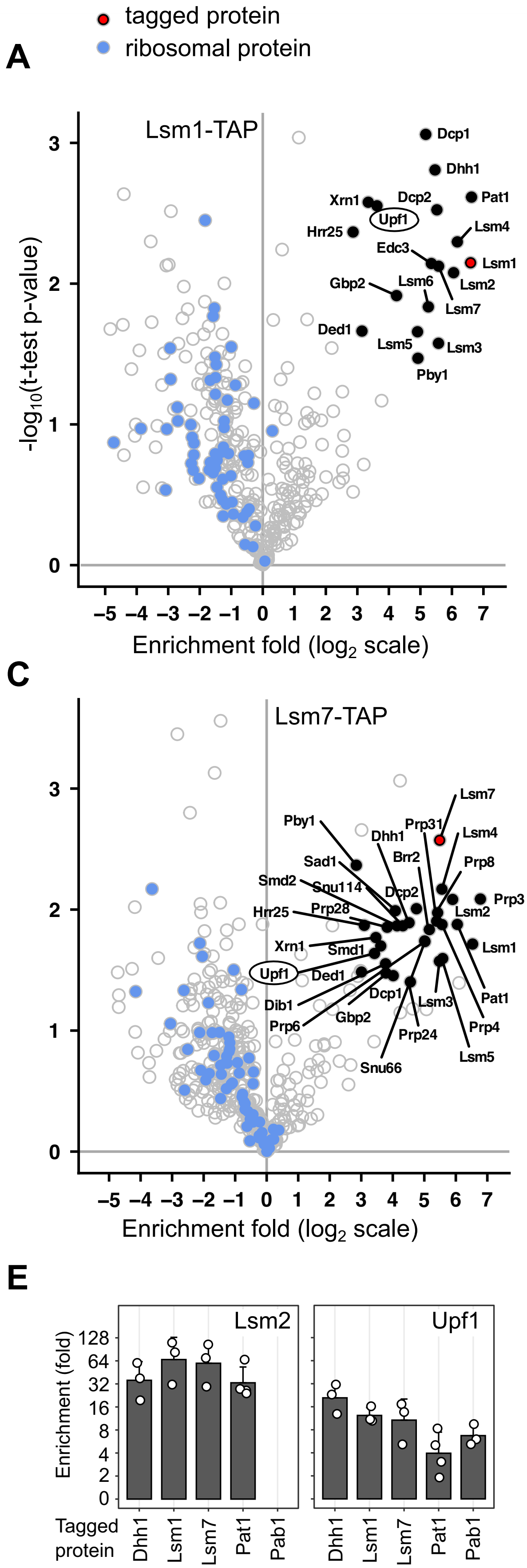

tagged protein
ribosomal protein
A
Lsm1-TAP
Dcp1
Dhh1
Pat1
Xrn1
Dcp2
Upf1
Hrr25
Lsm4
Edc3
Lsm1
Gbp2
Lsm6
Lsm2
Lsm7
Ded1
Lsm5
Lsm3
Pby1
-log10(t-test p-value)
Enrichment fold (log2 scale)
B
Pat1-TAP
Dhh1
Lsm1
Lsm2
Pat1
Nrp1
Lsm4
Lsm3
Lsm5
Lsm6
Ded1
Lsm7
Xrn1
Rbg1
Eap1
Upf1
Dcp2
Hrr25
Gbp2
Npl3
Edc3
C
Lsm7-TAP
Lsm7
Pby1
Dhh1
Prp31
Lsm4
Sad1
Brr2
Prp8
Smd2
Snu114
Dcp2
Prp3
Hrr25
Prp28
Lsm2
Xrn1
Smd1
Lsm1
Upf1
Ded1
Dib1
Dcp1
Lsm3
Pat1
Prp6
Gbp2
Prp24
Prp4
Lsm5
Snu66
D
Dhh1-TAP
Lsm1
Dhh1
Lsm5
Mss116
Lsm3
Nop1
Dcp2
Eap1
Scd6
Lsm6
Ded1
Psp2
Gbp2
Xrn1
Dcp1
Hek2
Lsm4
Pab1
Upf1
Pby1
Sbp1
Prp43
Lsm2
Rpa49
Ald5
Npl3
Pbp1
Nop58
Hrr25
Ebs1
Nop4
Lsm7
Mam33
Sro9
Lsm12
Cbf5
Imd3
Utp22
Nop56
E
Lsm2
Upf1
Enrichment (fold)
Tagged protein
Dhh1
Lsm1
Lsm7
Pat1
Pab1

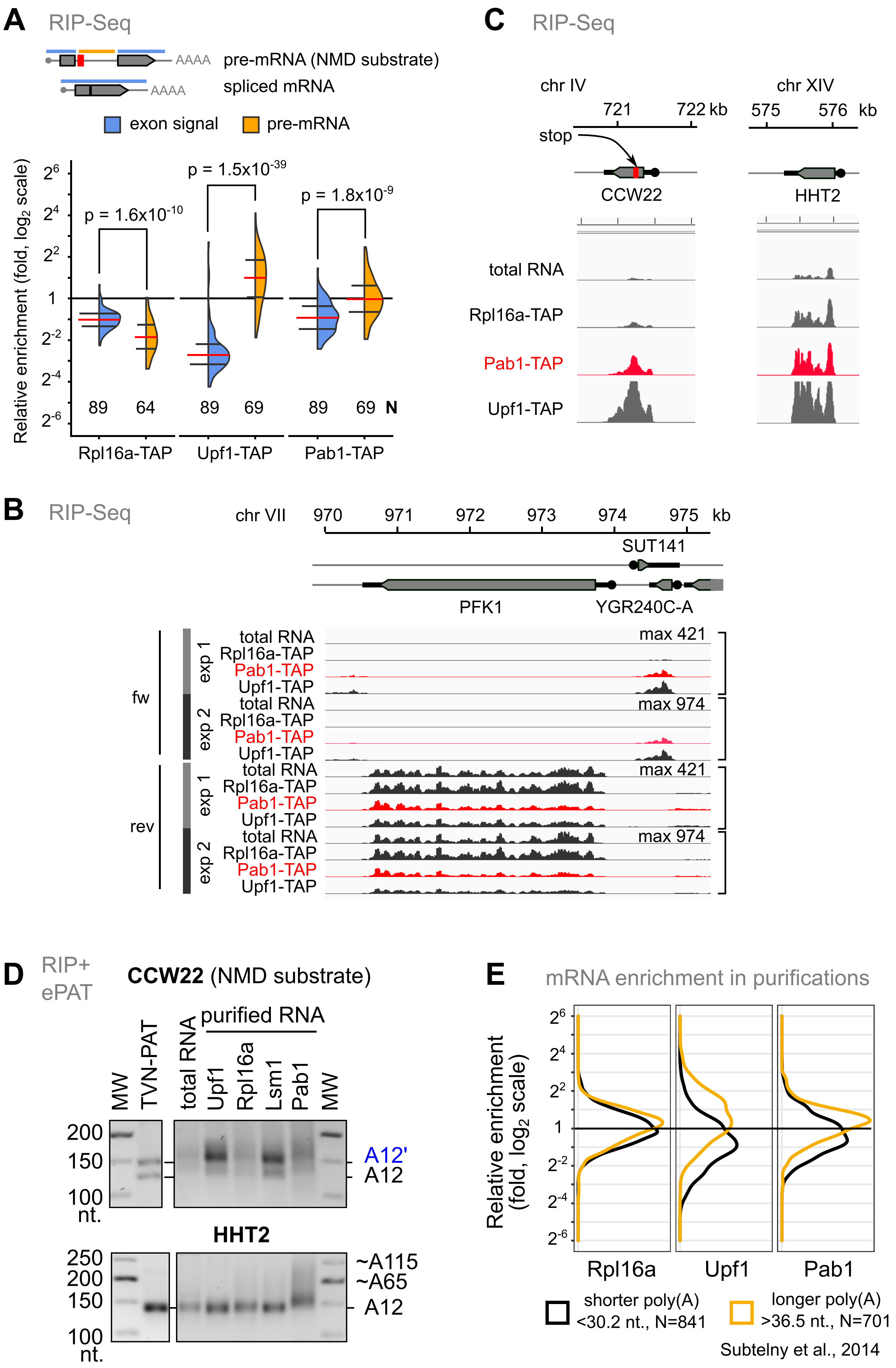
A
RIP-Seq
AAAA pre-mRNA (NMD substrate)
AAAA spliced mRNA
exon signal
pre-mRNA
Relative enrichment (fold, log2 scale)
p = 1.6x10-10
p = 1.5x10-39
p = 1.8x10-9
89 64 89 69 89 69 N
Rpl16a-TAP
Upf1-TAP
Pab1-TAP
C
RIP-Seq
chr IV
721 722 kb
stop
CCW22
chr XIV
575 576 kb
HHT2
total RNA
Rpl16a-TAP
Pab1-TAP
Upf1-TAP
B
RIP-Seq
chr VII 970 971 972 973 974 975 kb
SUT141
PFK1
YGR240C-A
fw
rev
exp 1
exp 2
total RNA
Rpl16a-TAP
Pab1-TAP
Upf1-TAP
max 421
max 974
D
RIP+ ePAT
CCW22 (NMD substrate)
purified RNA
MW
TVN-PAT
total RNA
Upf1
Rpl16a
Lsm1
Pab1
MW
200
150
100
nt.
A12'
A12
HHT2
250
200
150
100
nt.
~A115
~A65
A12
E
mRNA enrichment in purifications
Relative enrichment (fold, log2 scale)
Rpl16a
Upf1
Pab1
shorter poly(A) <30.2 nt., N=841
longer poly(A) >36.5 nt., N=701
Subtelny et al., 2014

 © The Author(s)

◀ **Figure EV2. Unstable mRNAs are associated with Pab1.**

(**A**) Relative enrichment of pre-mRNA for ribosomal protein genes (intron signal, orange) in comparison with the spliced mRNA for the same category (exon signal, blue). The red horizontal line indicates the median of the enrichment values, with the first and third quartiles indicated. The p-values correspond to a Welch two sample t-test, *N* is the number of values in each category. (**B**) Example of signal intensity for RNAs associated with Pab1-TAP, Rpl16a-TAP, or Upf1-TAP for a region of the yeast genome that corresponds to SUT141, an unstable RNA, in comparison with the PFK1 stable mRNA (transcribed in proximity but from the opposite strand). The vertical scale is the same for the samples of the same replicated experiment. Pab1 associated RNA signal is depicted in red. The image was obtained with the Integrated Genome Viewer, IGV. (**C**) Similar to (**B**) for the regions of transcripts CCW22, and NMD substrate (left), and HHT2 (right). (**D**) Estimation of poly(A) tail distribution for CCW22 and HHT2 in the RNA fractions enriched with Upf1, Rpl16a, Lsm1, and Pab1. The TVN-PAT lane corresponds to a control experiment with an anchored 3′ primer. For CCW22, two 3′ ends were detected (noted A12, and A12′). (**E**) The distribution of the enrichment values for two extreme categories of mRNA, with long (orange) or short average poly(A) tails (black) was depicted for the purified samples in association with Rpl16a-TAP, Upf1-TAP, and Pab1-TAP. A Wilcoxon ranks sum test with continuity correction comparing the distribution of relative enrichment values for long poly(A) tails mRNA versus short poly(A) tails, led to the following p values (for a minimum 25% increase): 0.9997 for Rpl16a, less than 2×10^{-16} for Upf1 and 1.9×10^{-8} for Pab1.

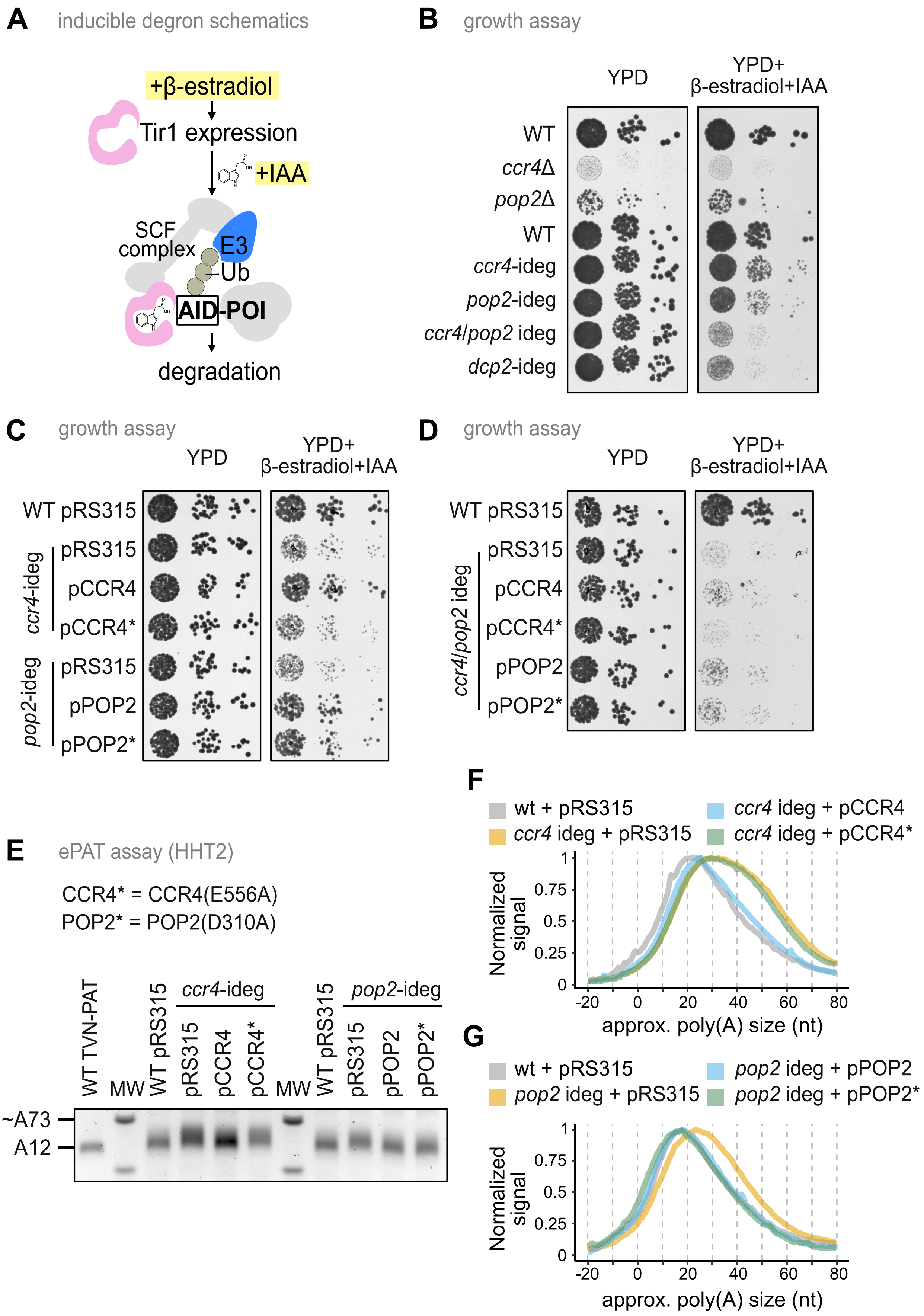
A
inducible degron schematics
+β-estradiol
Tir1 expression
+IAA
SCF complex
E3
Ub
AID-POI
degradation
B
growth assay
YPD
YPD+ β-estradiol+IAA
WT
ccr4Δ
pop2Δ
WT
ccr4-ideg
pop2-ideg
ccr4/pop2 ideg
dcp2-ideg
C
growth assay
YPD
YPD+ β-estradiol+IAA
WT pRS315
ccr4-ideg
pRS315
pCCR4
pCCR4*
pop2-ideg
pRS315
pPOP2
pPOP2*
D
growth assay
YPD
YPD+ β-estradiol+IAA
WT pRS315
ccr4/pop2 ideg
pRS315
pCCR4
pCCR4*
pPOP2
pPOP2*
E
ePAT assay (HHT2)
CCR4* = CCR4(E556A)
POP2* = POP2(D310A)
WT TVN-PAT
MW
WT pRS315
ccr4-ideg
pRS315
pCCR4
pCCR4*
MW
WT pRS315
pop2-ideg
pRS315
pPOP2
pPOP2*
~A73
A12
F
wt + pRS315
ccr4 ideg + pRS315
ccr4 ideg + pCCR4
ccr4 ideg + pCCR4*
Normalized signal
approx. poly(A) size (nt)
G
wt + pRS315
pop2 ideg + pRS315
pop2 ideg + pPOP2
pop2 ideg + pPOP2*
Normalized signal
approx. poly(A) size (nt)

 © The Author(s)

Figure EV3. An inducible degron system for deadenylases.

(**A**) Schematics of the developed inducible degron system in which *O. sativa* Tir1 expression is under the control of an estrogen sensitive promoter. In the presence of the plant auxin hormone indole-3-acetic acid (IAA), Tir1 targets a E3 ubiquitin ligase to the AID domain fused to the protein of interest (POI) for its rapid proteasome degradation. (**B**) Serial dilution plate growth assay for deletion and inducible degron (ideg) strains affecting deadenylases of the CCR4/NOT complex and the decapping enzyme Dcp2. Growth on rich medium (YPD, left) was compared with growth on rich medium containing β-estradiol and IAA (right). (**C**) Similar to (**B**) for the strains depleted for Ccr4 or Pop2 that were transformed with a control plasmid (pRS315), pRS315-CCR4 or pRS315-POP2. The asterisks indicates mutated versions of Ccr4, E556A, and Pop2, D310A. (**D**) Similar with (**C**), but for the concomitant depletion of Ccr4 and Pop2. (**E**) Changes in the poly(A) tail size of HHT2 measured using ePAT in the strains presented in panel (**C**). The TVN-PAT lane indicates the relative position of the A12 RNA. (**F**) Poly(A) size profile for HHT2 under Ccr4 depletion conditions was extracted from panel (**E**) and normalized to the signal in each lane. (**G**) Similar to (**F**), but for the depletion of Pop2.

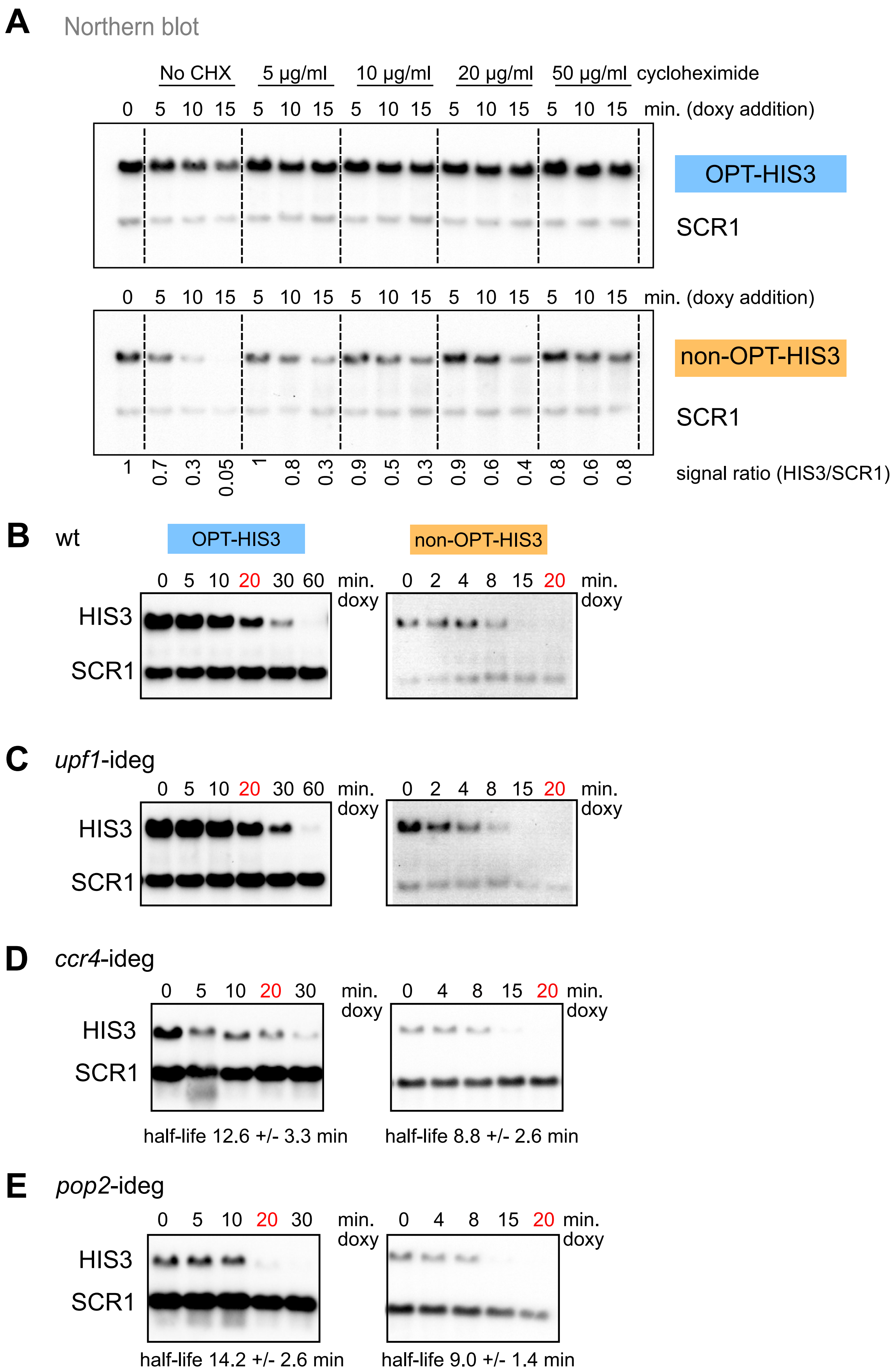
A
Northern blot
No CHX
5 µg/ml
10 µg/ml
20 µg/ml
50 µg/ml
cycloheximide
0 5 10 15 5 10 15 5 10 15 5 10 15 5 10 15 min. (doxy addition)
OPT-HIS3
SCR1
0 5 10 15 5 10 15 5 10 15 5 10 15 5 10 15 min. (doxy addition)
non-OPT-HIS3
SCR1
1 0.7 0.3 0.05 1 0.8 0.3 0.9 0.5 0.3 0.9 0.6 0.4 0.8 0.6 0.8
signal ratio (HIS3/SCR1)
B
wt
OPT-HIS3
non-OPT-HIS3
0 5 10 20 30 60 min. doxy
0 2 4 8 15 20 min. doxy
HIS3
SCR1
C
upf1-ideg
0 5 10 20 30 60 min. doxy
0 2 4 8 15 20 min. doxy
HIS3
SCR1
D
ccr4-ideg
0 5 10 20 30 min. doxy
0 4 8 15 20 min. doxy
HIS3
SCR1
half-life 12.6 +/- 3.3 min
half-life 8.8 +/- 2.6 min
E
pop2-ideg
0 5 10 20 30 min. doxy
0 4 8 15 20 min. doxy
HIS3
SCR1
half-life 14.2 +/- 2.6 min
half-life 9.0 +/- 1.4 min

 © The Author(s)

Figure EV4. Degradation of OPT-HIS3 and non-OPT-HIS3 reporter mRNAs depends on translation and decapping.

(**A**) Dose-dependent effect of the translation inhibitor cycloheximide (from 0 to 50 µg/ml) was tested by Northern blotting for optimal (upper panel) and non-optimal (lower panel) HIS3 reporters. SCR1 was used as a loading control. Degradation rates for optimized HIS3 (left panel) and non-optimal HIS3 (right panel) were visualized by Northern blot (HIS3 reporter signal) in comparison with an SCR1 control. (**B**) Similar to (**A**), in a wild type strain. (**C**) Similar to (**A**) after depletion of Upf1. (**D**) Similar to (**A**) after depletion of Ccr4. (**E**) Similar to (**A**) after depletion of Pop2. For each situation, the estimated half-life and the 95% confidence interval obtained from RT-qPCR and the results are indicated.

© The Author(s)

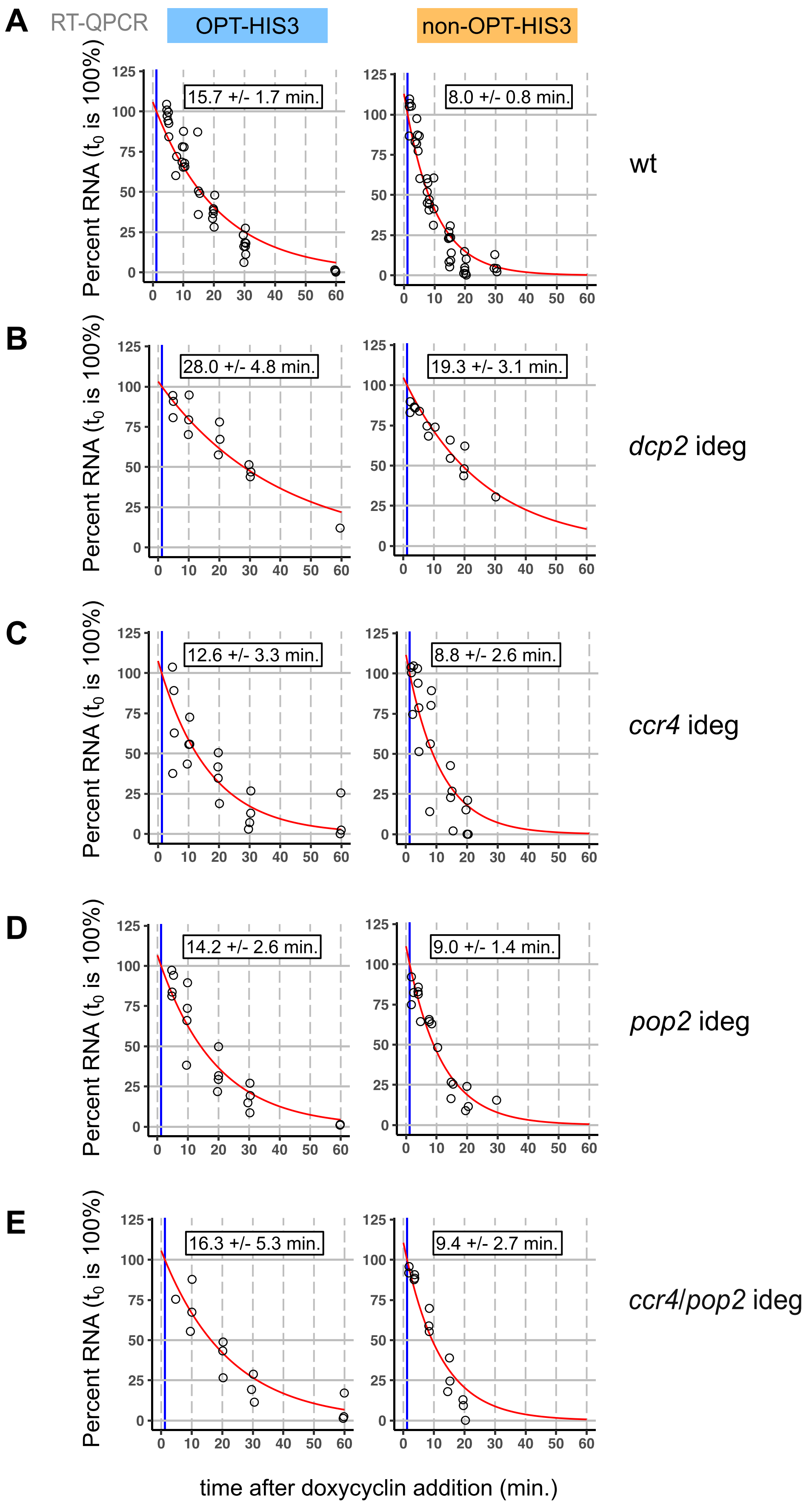
A
RT-QPCR
OPT-HIS3
non-OPT-HIS3
15.7 +/- 1.7 min.
8.0 +/- 0.8 min.
wt
B
28.0 +/- 4.8 min.
19.3 +/- 3.1 min.
dcp2 ideg
C
12.6 +/- 3.3 min.
8.8 +/- 2.6 min.
ccr4 ideg
D
14.2 +/- 2.6 min.
9.0 +/- 1.4 min.
pop2 ideg
E
16.3 +/- 5.3 min.
9.4 +/- 2.7 min.
ccr4/pop2 ideg
Percent RNA (t0 is 100%)
0
25
50
75
100
125
0 10 20 30 40 50 60
time after doxycyclin addition (min.)

 © The Author(s)

Figure EV5. Half-life of reporter RNAs as measured by RT-qPCR.

(**A**) The half-life of HIS3 reporter, codon-optimized (left panel) or non-optimized (right panel), was measured by RT-qPCR following transcription block with doxycyclin. For each decay experiment, the values were normalized ot time 0, considered to represent 100%. Indicated estimates correspond to half-life in minutes with a 95% confidence interval. All the experiments were performed independently at least three times. (**B**) Similar to (**A**), but after depletion of Dcp2. (**C**) Similar to (**A**), but after depletion of Ccr4. (**D**) Similar to (**A**), but after depletion of Pop2. (**E**) Similar to (**A**) but after concomitant depletion of Ccr4 and Pop2.
